# Adenoidectomy: Our Approach

**DOI:** 10.1155/2010/628969

**Published:** 2010-06-30

**Authors:** Mohnish Grover

**Affiliations:** Department of ENT, SMS Medical College and Hospital, Jaipur, India

Dear Sir, it was a pleasure reading the article “Transoral endoscopic adenoidectomy” by A. El-Badrawy and M. Abdel-Aziz published in Volume 2009 (2009) of this journal (Article ID 949315). Adenoidectomy has been an old surgery and has been done with various techniques [1]. The technique described in this article is definitely better than conventional technique of adenoidectomy. 

However, I feel that in this era of use of microdebrider for sinus and laryngeal surgery, endoscopic assisted powered adenoidectomy is a natural progression of this technology to allow a more complete adenoidectomy [2, 3]. The powered microdebrider XPS 3000 (Medtronic Xomed, Inc., Jacksonville, FL) is ideally suited for powered adenoidectomy. The blade tip (RADenoid 40, 4 mm, Medtronic Xomed, Inc.) has an outer windowed sheath surrounding an inner rotating blade, connected to a standard inline continuous suction-irrigation system through the handpiece. The blade is angled at 40 degrees which makes it very convenient to use. It has a length of 11 cms. The device is used at a setting of 1500 rpm in oscillating mode. 

I use a 2.7 mm 0 degree endoscope to visualise the area thereby providing a more direct and complete view. Patient is taken up under general anesthesia with orotracheal intubation. The nose is decongested well with help of saline-adrenaline nasal packs as for FESS. A Boyle-Davis mouth gag is inserted and suspended on Draffin bipod. The microdebrider is inserted into the nasopharynx transorally. Nasal endoscope is inserted through the nose to have a head on view of the adenoid tissue. The surgeon directs the blade window towards the adenoid tissue to be removed; this is drawn in by the vacuum, and the rotating blade then shaves the tissue. The resection begins at choana and progresses inferiorly and posteriorly with use of a side-to-side motion until the desired amount of adenoid tissue has been removed [4]. This procedure prevents any injury to surrounding structures and decreases the chances of any residual/recurrence. The blood loss, operative time, and chances of injury to Eustachian tube are also reduced. 

We have used this technique in 157 cases with no complications so far. I strongly believe that this procedure should be considered as the standard procedure for adenoidectomy, as a head on view of the site of surgery gives better information about the amount and areas from which the tissue needs to be removed than an angled view from the oral cavity. The only issue involved could be the availability of microdebrider, but I think it is available in all the tertiary care centres and in times to come it would replace the usual cold instruments.
